# The Emerging Roles of CCN3 Protein in Immune-Related Diseases

**DOI:** 10.1155/2021/5576059

**Published:** 2021-05-18

**Authors:** Linan Peng, Yingying Wei, Yijia Shao, Yi Li, Na Liu, Lihua Duan

**Affiliations:** ^1^Department of Rheumatology and Clinical Immunology, Jiangxi Provincial People's Hospital Affiliated to Nanchang University, Nanchang, China; ^2^School of Medicine, Xiamen University, Xiamen, China; ^3^Department of Rheumatology and Immunology, Tongji Hospital, Tongji Medical College, Huazhong University of Science and Technology, China

## Abstract

The CCN proteins are a family of extracellular matrix- (ECM-) associated proteins which currently consist of six secreted proteins (CCN1-6). CCN3 protein, also known as nephroblastoma overexpressed protein (NOV), is a member of the CCN family with multiple biological functions, implicated in major cellular processes such as cell growth, migration, and differentiation. Recently, CCN3 has emerged as a critical regulator in a variety of diseases, including immune-related diseases, including rheumatology arthritis, osteoarthritis, and systemic sclerosis. In this review, we will briefly introduce the structure and function of the CCN3 protein and summarize the roles of CCN3 in immune-related diseases, which is essential to understand the functions of the CCN3 in immune-related diseases.

## 1. Introduction

The name of the CCN family derived from the acronym of the first three discovered proteins, namely, cysteine-rich protein 61 (CYR61, CCN1), connective tissue growth factor (CTGF, CCN2), and nephroblastoma overexpressed (NOV, CCN3) [1–3]. The other three members, WISP1 (CCN4), WISP2 (CCN5), and WISP3 (CCN6), are considered Wnt-inducible secreted proteins, participating in the Wnt signaling pathway. All the CCN members (except for CCN5) share four conservative homologous domains following an N-terminal secretion signal-peptide: insulin growth factor-binding protein (IGFBP), von Willebrand factor type C (vWC), thrombospondin type 1 repeat (TSP-1), and carboxy-terminal knot domain (CT) [4]. CCN5 is the particular one that lacks the CT domain. The CCN members are matricellular proteins, and their main function is to facilitate the interaction between cells and extracellular matrix (ECM) rather than maintaining structural stability. As secreted proteins, CCNs have crucial roles in multiple biological processes through combination with heparan sulfate proteoglycan (HSPG), different types of integrators, and other noncanonical receptors [5–8]. CCNs promote the adhesion, mitosis, and migration of human fibroblasts through interaction with integrins *α*6*β*1, *α*v*β*3, *α*v*β*5, and HSPG which also play a critical role in the process of mediating fibroblast adhesion [4, 9, 10]. Moreover, it has been shown that adhesion of CCN1 to fibroblasts can induce apoptosis, while adhesion to endothelial cells can promote cell survival [11]. CCN2 can also bind to integrins *α*v*β*3 and HSPG, which induces the rat activated hepatic stellate cell adhesion [12]. Although CCN3 shares homologous structures with CCN1 and CCN2, it is quite different in some biological functions. For example, CCN3 is not necessary for embryonic development compared to CCN1 and CCN2 [13]. However, CCN3 can induce angiogenesis through the ligands *α*v*β*3 and *α*5*β*1 [14].

CCN3/NOV (nephroblastoma overexpressed) was first isolated from the myeloblastosis-associated virus- (MAV-) induced nephroblastoma in day-old chicks [3]. In human early embryonic development, CCN3 is widely expressed in the derivative of all three germ layers [15]. In adult mammals, high CCN3 expression is observed in endotheliocytes, smooth muscle cells, fibroblasts, and chondrocytes [14, 16, 17]. Besides, Dombrowski et al. first discovered that CCN3 can be produced by regulatory T cells (Treg) [18]. CCN3 exerts biological functions through binding different receptors. It is reported that CCN3 could directly act on endothelial cells by binding integrins *α*v*β*3 and *α*5*β*1 to promote cell adhesion, migration, and cell survival in vitro and induce angiogenesis, while these observations can be blocked by CCN3 inhibitors [14]. Furthermore, the aberrant expression of CCN3 is also involved in fibrosis and cancers [19, 20]. CCN3 may inhibit the activity of NOTCH1 by binding to the extracellular domain of NOTCH1 in CML [21], and the CCN3 protein secreted by prostate cancer (PCa) could recruit macrophages and promote their differentiation into the M2 phenotype [22]. CCN2 is the most well-known fibrosis-related protein in the CCN protein family. At present, the anti-CCN2 antibody (FG-3019) has been used in clinical trials of patients with idiopathic pulmonary fibrosis and has achieved significant therapeutic effects [23]. Barbe et al. found that in animal fibrosis models, FG-3019 treatment can increase the expression level of CCN3 in muscles [24]. CCN3 has been shown as a negative regulator of CCN2 to antagonize the fibrogenesis effect of CCN2 and further to inhibit the progression of fibrosis [25].

Immune-mediated diseases refer to a group of diseases characterized by dysregulated immune responses, eventually leading to the damage of cells, tissues, and even organs. Several articles reported that CCN3 is involved in the regulation of immune cell function, such as the regulation of Treg and hematopoietic stem cell function, while other CCN family molecules are rarely reported in these years [18, 26, 27]. Furthermore, it has been found that the abnormal level of CCN3 is connected with immune regulation and immune-mediated diseases. Although the precise mechanism remains unclear, the insight into CCN3-mediated biological regulation could allow us a better understanding of the emerging role of CCN3 in immune-mediated diseases.

## 2. CCN3 in Pathophysiological Disorders

### 2.1. The Role of CCN3 in Endothelial Cell Function

It has been demonstrated that CCN3 maintains cardiovascular homeostasis by regulating VSMC and endothelial cell function [14, 16]. Lin et al. found that purified recombinant human CCN3 could mediate endotheliocyte adhesion through integrins *α*v*β*3, *α*6*β*1, and *α*5*β*1. Meanwhile, CCN3 can mediate endothelial cell migration as a ligand of *α*5*β*1 and *α*v*β*3 [14]. In addition, CCN3 exerted an inhibitory effect on VSMC proliferation and migration to resist neointimal hyperplasia. Further study found that CCN3 upregulated the cyclin-dependent kinase inhibitors p15 and p21 partly through the Notch signaling pathway independently of TGF-*β* signaling [16]. To further explore the function of CCN3 in regulating endothelial inflammation, Lin et al. found that KLF2, a factor that inhibited endothelial proinflammation progress, could increase the expression of CCN3 in HUVECs. Next, by exposing HUVECs infected with adenovirus-CCN3 to TNF-*α* or IL-1*β*, the adhesion molecules and VCAM-1 were strongly inhibited. On the contrary, knockdown of CCN3 markedly enhanced VCAM-1 expression induced by TNF-*α* stimulation. Mechanically, they found that CCN3 exerted a negative effect on NF-*κ*B accumulation induced by inflammatory cytokines [28]. These observations suggest that CCN3 played a vital role in regulating the endothelial cell function.

### 2.2. The Role of CCN3 in Fibrosis

The process of fibrosis involves a series of sequential and complex steps including the transient activation of fibroblasts, the proliferation of fibroblasts, and the production of excessive extracellular matrix (ECM), which is regulated by numerous cytokines such as transforming growth factor (TGF) and platelet-derived growth factor (PDGF) [29]. Studies have shown that all CCN family members, as matricellular protein, are related to fibrosis [30–33]. CCN2, as the downstream effector of TGF-*β*, has been implicated in the process of the initial period of fibrosis. Conversely, CCN3 could counter the downstream of the TGF-*β* signal pathway to inhibit fibrosis. CCN3 and CCN2 act as a yin and yang regulator to adjust the fibrosis development [34].

A previous study has shown that TGF-*β* regulates CCN2 function through MEK1, YAP1, or TAK1, while inhibiting these molecules will not affect the role of TGF-*β* on the CCN3 expression in human skin fibroblasts. Therefore, it is believed that the regulation of CCN3 was suppressed by some unknown factors [35]. The previous fibrosis models in vitro and in vivo have proved that CCN3 is a negative regulator of CCN2 and able to block the accumulation of ECM [25, 36]. In the recent TGF-*β* induced fibrotic models of renal fibroblasts, the increased expression of CCN2 and depressed level of CCN3 were detected in the conditional media [37]. In the chronic fibrosis induced by muscle overuse, researchers observed that the CCN3 expression was improved following the treatment of the CCN2 inhibitor (FG-3019) [24]. Although it was later found that CCN3 did not have a noticeable effect to attenuate the liver fibrosis due to hepatocyte apoptosis which occurred simultaneously [38], the correlations between CCN3 and antifibrosis actions have been identified in a variety of diseases such as chronic overuse muscle fibrosis, glomerular and tubulointerstitial renal fibrosis, and systemic sclerosis [24, 39–41]. Thus, a hypothesis was proposed which also paid attention to the fact that the ratio of CCN2 : CCN3 and balancing the interaction of CCN proteins might be an important measure for the treatment of fibrosis [19].

### 2.3. The Role of CCN3 in Tumor Proliferation

It is clear that the levels of CCN3 are abnormal in some certain tumors. However, the role of CCN3 in the tumor is exceedingly complex, because the functions of CCN3 have been shown to differ in various types of malignancies [42]. Maillard et al. found that CCN3 was expressed predominantly in prostate cancer cell lines as well as the lymph node metastases compared with normal prostate epithelial cells [43], and similar results were obtained in cervical cancer, bone malignancies, and benign adrenocortical tumors [44–47]. In addition, Chen et al. first reported that prostate cancer- (PCa-) secreted CCN3 had the capacity to recruit macrophages and promote their differentiation to an M2 phenotype, and macrophage migration was induced by conditioned media (CM) from various PCa cells and was inhibited by an anti-CCN3 neutralizing antibody. These functions of CCN3 might be associated with PCa-derived CCN3-induced focal adhesion kinase (FAK)/AKT/NF-*κ*B signaling, which also lead to increased VEGF expression and increased tube formation in endothelial progenitor cells [22]. These observations suggested that CCN3 was associated with cancer staging and prognosis as well as contributing to tumorigenesis or metastasis formation On the other hand, CCN3 can also negatively regulate some other kinds of tumor growth, such as melanoma, glioblastoma, and chronic myeloid leukemia [42, 48, 49]. The decreased expression of CCN3 was investigated in the invasion melanoma cells, and CCN3 transduction lessened the invasion through restraining the MMP-2 and MMP-9 activities [48]. The cell growth of the K562 CML cell line stably transfected with CCN3 was significantly decreased, especially the number of cells in the subG0 phase increased. Furthermore, the apoptosis of K562 cells treated with CCN3 and imatinib was enhanced, suggesting that CCN3 may affect the process of cell mitosis and enhance imatinib-induced cell apoptosis in CML [49]. Consistently, CCN3 could act as an antiproliferative protein by influencing the cell cycle of glioblastoma cells [50].

## 3. The Roles of CCN3 Protein in Immune-Related Diseases

### 3.1. Rheumatoid Arthritis (RA)

Previous studies has shown that CCN1, CCN2, CCN4, and CCN5 are highly expressed in OA and RA knee cartilages, while CCN3 and CCN6 can hardly be detected in OA and RA cartilages. However, the CCN3 gene was highly expressed in OA and RA synovial samples compared with normal joint tissues [51]. RA is a chronic systemic autoimmune disease that eventually leads to cartilage and bone destruction and joint dysfunction. However, the role of CCN3 in rheumatoid arthritis (RA) remains elusive. Recently, our data showed that the serum CCN3 level of RA patients was obviously increased in RA, and the immunohistological analysis revealed a considerable increased deposition of CCN3 in the joint tissues from RA patients, but not in the control tissues from OA patients [52]. This may be due to the use of different antibodies in the test. IL-6 and TNF-*α* are critical inflammatory factors in the RA progression [53]; our data demonstrated that CCN3 positively connected with IL-6 expression but no statistical difference with TNF-*α* [52]. Our work suggested that CCN3 may serve as a biomarker for inflammation and disease activity in RA, but the mechanism of CCN3 remains to be deeply elucidated. Besides, the expression of CCN1 was also higher in RA but was inversely correlated with RA disease activity [54, 55]. Therefore, further studies on the function of CCN proteins are needed to resolve the specific regulatory mechanism of CCN proteins in the development of RA.

### 3.2. Osteoarthritis (OA)

OA, a major clinical problem among the ageing population, is characterized by articular cartilage degeneration and synovial inflammation. CCN3 was highly expressed in articular chondrocytes in the normal rat articular cartilage [17], but it has an apparent decline in a monoiodoacetic acid- (MIA-) induced osteoarthritic model [56]. Consistently, another research also confirmed that the CCN3 expression was reduced in the cartilage tissue of OA patients and OA rat models [57]. These data suggested that the expression of CCN3 might be a protective role in cartilage degeneration. Of note, exogenous recombinant CCN3 administration increased the accumulation of proteoglycan and the expression of tenascin-C and lubricin, protected the damage of articular cartilage surface, positively modulated chondrogenesis, and attenuated the progress of OA [56]. Furthermore, recombinant CCN3 or CCN3 overexpression could also ameliorate IL-1*β*-induced osteoarthritis response by reducing extracellular matrix catabolism and inducing cartilage protection in vitro via decreasing the level of HMGB1, reversing the increase of MMP, inhibiting the activation of PI3K/AKT/mTOR pathway, and promoting cell autophagy [57]. However, CCN3 dramatically suppressed the proliferation and activity of osteoblast and has an inhibitory effect on osteoblast differentiation by its involvement of the BMP and Notch signaling pathways, and higher phosphorylation of Smad1/5 was observed in CCN3 knockout mice [58, 59]. CCN3 positively regulates articular chondrocytes but inhibits osteoblast differentiation and acts as an inhibitor of bone regeneration. Therefore, the mechanisms of CCN3 in the bone metabolism need further to be explored.

### 3.3. Glomerulonephritis (GN)

Most of GN are thought to be immune mediated with abnormal regulation of both humoral immunity and cellular immunity, which cause the different sites of glomerular injury such as endothelial cell and mesangial area and result in various histopathological alterations including fibrosis, mesangial cell proliferation, and glomerular sclerosis [60]. Recently, CCN3 was suggested to play a crucial role in the development of some certain types of glomerulonephritis. It was shown that CCN3 could inhibit the fibrotic pathway by reducing the TGF-*β*-stimulated CCN2 expression and blocking the accumulation of extracellular matrix (ECM) such as collagen type I [25], which was further confirmed an in vitro model of diabetic renal fibrosis [36]. In consistency, exogenous recombinant CCN3 treatment dramatically downregulated the fibrosis-related factor (CCN2, Col1a2, TGF-*β*1, and PAI-1) mRNA in the kidney cortex of diabetes nephritis [40]. Similar observations were investigated in the culture of human mesangial cells; exogenous rCCN3 effectively controlled ECM formation and improved the TGF-*β* induced MMP expression [61]. Additionally, Roeyen et al. also found that CCN3 could act as an endogenous inhibitor of mesangial cell growth and a modulator of PDGF-induced mitogenesis in vitro [62]. In the experimental vascular proliferative nephritis model, the expression of glomerular CCN3 was increased in accordance with the decreased proliferation of mesangial cells. Furthermore, the proangiogenic and antiangiogenic effects of CCN3 in experimental glomerulonephritis have been determined [63]. Their observations indicate that CCN3 contributes to repairing glomerular endothelial injury and mesangial proliferation changes. Therefore, the CCN3 protein can be considered a potential therapeutic target for glomerulonephritis. However, further studies should be explored because the regulation of CCN3 in immune response during the development of nephritis remains unknown.

### 3.4. Metabolic Diseases

Type 2 diabetes mellitus (T2MD) has not been considered a typical immune-related disease; however, the disorder of the immune system in T2MD have already been found in adipose tissue, the liver, pancreatic islets, the vasculature, and circulating leukocytes which leads to insulin resistance and inflammation eventually. Recent investigation shows that serum CCN3 correlated positively with adiposity-related parameters and insulin resistance indices, which is the first study to focus on the serum concentration of CCN3 in newly diagnosed T2MD (nT2MD) in humans [64], and a strong relationship between plasma CCN3 and obesity was also detected by measuring hundreds of adults suffering from hyperlipidemia and/or receiving lipid-lowering treatment and/or having a high BMI (>30 kg/㎡) [65]. Consistently, it was shown that the CCN3^−/−^ mice gained less body weight and improved the glucose tolerance and insulin sensitivity along with lower inflammation in the adipose tissue compared with wild-type controls when facing high-fat diet, although insulin production remained roughly in equal level. Interestingly, the absence of CCN3 led to a significant decrease expression of several proinflammatory cytokines and chemokines in the adipose tissue, which was associated with a change in the macrophage profile (M1-like to M2-like) [66]. CCN3 can also affect the phagocytosis of macrophages; macrophage from CCN3^−/−^ mice leads to the increase of oxLDL uptake and foam cell formation through upregulated CD36 and SRA1 expressions. At the atherosclerotic lesions, Apoe^−/−^ with CCN3 depletion increased their lipid plaque formation, macrophage infiltration, and the expression of monocyte chemotactic protein 1 compared to Apoe^−/−^mice in vivo with high-fat feed [67]. Additionally, CCN3 has been found to be a new target for the transcription factor, FoxO1, which is a prominent mediator of insulin signaling in pancreatic *β*-cells. Activation of FoxO1 increased the expression of CCN3 in transgenic mice. On the other hand, CCN3 could inhibit the proliferation of *β*-cells, leading to the decline of insulin secretion in pancreatic *β*-cells [68]. Taken together, CCN3 antagonists can be regarded as a potential therapeutic strategy for T2MD. However, most studies have focused on the association between CCN3 and T2MD, while few have reported type 1 diabetes mellitus (T1DM). It is noteworthy that further studies are needed to explore the mechanistic details of CCN3 in T1MD.

### 3.5. Multiple Sclerosis (MS)

It have been demonstrated that CCN3 expression could be detected in the nervous system [15, 69]; the roles of CCN3 in the central nervous system also have gained a lot of attention. Dombrowski et al. firstly reported that CCN3, as a growth-regulating protein, was produced by regulatory T cells (Treg). Anti-CCN3 antibody treatment or depleting CCN3 from Treg-conditioned media could abolish or inhibit the Treg-induced oligodendrocyte-differentiating effect and promyelinating effect. Furthermore, the treatment with recovered CCN3 significantly strengthens brain slice myelination [18]. Subsequent further study has found that increased CCN3 expression was observed in the progression of myelination in vivo. However, there is no significant difference between CCN3 knockout and wild-type control mice in the proliferation and differentiation of oligodendrocyte progenitor cell. Therefore, it is speculated that CNS cells cocultured with glial cells are affected by CCN3, which indirectly affects the differentiation of OPC [70]. A recent data from clinical samples showed that the serum CCN3 level was positively correlated with the CSF CCN3 level. In addition, the CCN3 mRNA expression was higher in peripheral immune cells (PBMC) of MS patients compared with the healthy control group [71]. Therefore, the myelin regeneration function of Tregs and CCN3 can be offered as potential encouraging treatment prospects for multiple sclerosis, while further experiments should be performed to address the mechanism.

### 3.6. Systemic Sclerosis (SSc)

SSc is a chronic connective tissue disease characterized by diffuse or localized skin involvement, which is classified as an autoimmune rheumatic disease. Diffuse microangiopathy, inflammation, autoimmunity, and visceral and vascular fibrosis in multiple organs are mainly pathophysiologic processes of SSc [72]. CCN3 has been proved to be involved as part of the processes above, including antifibrosis and proangiogenesis effects [25, 63]. A study by Lemaire et al. showed that CCN3 plays a counterregulatory role in matrix formation by inhibiting the matrix assembly of fibrillary protein-1, providing a steady-state feedback mechanism for the control of extracellular matrix. These results are directly related to the early diffuse SSc skin [41]. Besides, the dermal capillary damage of the SSc patients' skin was associated with downregulation of CCN3 in dermal vessels and endothelial cells. Blocking CCN3 of human dermal microvascular endothelial cells (HDMECs) can inhibit angiogenesis, and HDMECs can promote angiogenesis of SSc HDMECs [73]. Actually, other CCN family members also participate in the pathological process of SSc [74]. In the bleomycin-induced model of skin scleroderma, the loss of CCN2 resulted in resistance to bleomycin-induced fibrosis, including the decrease of skin thickness, collagen production, and the loss of *α*-SMA-expressing myofibroblasts [75]. The previous studies have shown that CCN3 is a negative fibrosis of CCN2 and able to block the accumulation of ECM [25, 36]. Thus, CCN3 may be identified as a promising approach for SSc treatment.

## 4. Conclusion

Numerous basic studies on CCN3 have been explored in immune-related diseases; however, its role and mechanism in the pathogenesis of diseases remain elusive. In the current review, we tried to comprehensively summarize the contribution of CCN3 in the development of immune-mediated disease (Table 1). Of note, the biological effects of CCN3 are best known as the fibrosis inhibitor and proangiogenic factor, and these functions can be observed in many immune-related diseases. However, the regulation of CCN3 on immune cell function is only observed in certain cells, such as Treg and macrophages. These findings suggest that CCN3 might indirectly affect the immune cell function, which leads to the development of immune-related diseases. In addition, the receptors of CCN3 should be explored in the other immune cells. Previous studies almost focused on exploring the effects of CCN3 on animal models and in vitro experiment. Recent studies provide more and rich data from clinical samples [52, 64, 71, 73]. Although CCN3 targeted therapy has not been used in clinical immune diseases, we believe that the application of the anti-CCN2 antibody in clinical trials will contribute to further clinical research of CCN3 to a certain extent. As mentioned above, the CCN2 blocking antibody can increase the expression of CCN3 in the muscle and inhibit the progression of fibrosis in the model [24]. This also means that the CCN proteins have the same domain, and their functions show synergistic stimulation or inhibition to a certain extent. Therefore, further studies, particularly a clinical study, are required to fully understand the pathophysiological function of CCN3 in immune-related diseases, which helps to develop immunomodulatory therapeutics against the abnormal immune response.

## Figures and Tables

**Table 1:** Immune-related diseases influenced by CCN3.

Diseases	Mechanisms
Osteoarthritis (OA)	Decreases MMP, reduces extracellular matrix catabolism, and decreases the level of proinflammatory cytokines
Glomerulonephritis (GN)	Antifibrosis, inhibits mesangial cell growth, and inhibits the acceleration of ECM
Metabolic diseases	Inhibits the proliferation of pancreatic *β*-cells, regulates macrophage function, and regulates insulin signaling
Multiple sclerosis (MS)	Regulates Treg function and promotes myelin regeneration
Systemic sclerosis (SSc)	Antifibrosis, proangiogenesis, and controls extracellular matrix deposition
Rheumatoid arthritis (RA)	Decreases inflammatory cytokines

## Data Availability

No data were used to support this study.
